# New Statement about NRF2 in Amyotrophic Lateral Sclerosis and Frontotemporal Dementia

**DOI:** 10.3390/biom12091200

**Published:** 2022-08-29

**Authors:** Isabel Lastres-Becker, Eva de Lago, Ana Martínez, Javier Fernández-Ruiz

**Affiliations:** 1 Department of Biochemistry, School of Medicine, Institute Teófilo Hernando for Drug Discovery, Instituto de Investigaciones Biomédicas “Alberto Sols” UAM-CSIC, Universidad Autónoma de Madrid, Arturo Duperier 4, 28029 Madrid, Spain; 2 Centro de Investigación Biomédica en Red de Enfermedades Neurodegenerativas (CIBERNED), Instituto de Salud Carlos III, 28031 Madrid, Spain; 3 Instituto Universitario de Investigación en Neuroquímica, Departamento de Bioquímica y Biología Molecular, Facultad de Medicina, Universidad Complutense and Instituto Ramón y Cajal de Investigación Sanitaria (IRYCIS), 28040 Madrid, Spain; 4 Centro de Investigaciones Biológicas Margarita Salas, Consejo Superior de Investigaciones Científicas CSIC. Ramiro de Maeztu 9, 28040 Madrid, Spain

**Keywords:** ALS, FTD, FTLD, NRF2, neurodegeneration, NQO1, HO-1

## Abstract

Amyotrophic lateral sclerosis (ALS) and frontotemporal dementia (FTD) are related neurodegenerative disorders displaying substantial overlay, although there are substantial differences at the molecular level. Currently, there is no effective treatment for these diseases. The transcription factor NRF2 has been postulated as a promising therapeutic target as it is capable of modulating key pathogenic events affecting cellular homeostasis. However, there is little experimental evidence on the status of this pathway in both ALS and FTD. Therefore, in this work, we wanted to carry out an exhaustive analysis of this signaling pathway in both transgenic mouse models (ALS and FTD) and human samples from patients with sporadic ALS (sALS) versus controls. In samples from patients with sALS and in the transgenic model with overexpression of TDP-43^A315T^, we observed a significant increase in the NRF2/ARE pathway in the motor cortex and the spinal cord, indicating that NRF2 antioxidant signaling was being induced, but it was not enough to reach cellular homeostasis. On the other hand, in the transgenic FTD model with overexpression of the TDP-43^WT^ protein in forebrain neurons, a significantly decreased expression of NQO1 in the prefrontal cortex was seen, which cannot be attributed to alterations in the NRF2 pathway. Our results show that NRF2 signature is differently affected for ALS and FTD.

## 1. Introduction

Amyotrophic lateral sclerosis (ALS) and frontotemporal dementia (FTD), also referred to as frontotemporal lobar degeneration (FTLD), are two neurodegenerative diseases that are intrinsically associated with an overlay at clinical, neuropathological, and, in particular, genetic levels. As it is becoming more and more evident that both pathologies have so many points in common, they are currently considered to represent two ends of one disease spectrum, called the ALS–FTD spectrum [1,2,3]. From a genetic point of view, TARDBP, SQSTM1, VCP, FUS, TBK1, CHCHD10, and C9orf72 have been described as critical genetic players in these neurological conditions. TAR DNA-binding protein 43 (TDP-43), encoded by the *TARDBP* gene, has been found to be the major pathological protein of ALS as well as FTLD with ubiquitin-immunoreactive inclusions (FTLD-U) with or without ALS [4]. ALS is a fatal neurological disorder primarily affecting the motor system but in which extra-motor manifestations are increasingly accepted. In the motor cortex, brain stem nuclei, and anterior horn of the spinal cord, there is a loss of upper and lower motor neurons, which gives rise to progressive muscle weakness and wasting [5,6]. The disease typically begins as muscle weakness in a limb, which progresses to generalized weakness and paralysis of respiratory muscles, eventually leading to death due to respiratory failure. Approximately 10% of all ALS cases have a family genetic link to the disease, while the remaining 90% are sporadic. Despite this diverse etiology of the disease, 97% of patients display a common phenotype in disease-affected tissues, namely, the deposition of TDP-43 protein [7]. On the other hand, frontotemporal dementias (FTDs) encompass several disorders commonly characterized by a progressive decline in behavior or language associated with degeneration of the frontal and anterior temporal lobes, particularly in patients before the age of 65 years [8]. FTD is characterized by the deposition of aberrant inclusions of TDP-43, FUS, and TAU proteins. TDP-43 and FUS pathology are present in most patients with FTD, accounting for approximately 50% and 5%, respectively [8]. However, approximately 45% of FTD patients show FTD-associated mutations on *MAPT*, the TAU-coding gene, such as P301L [9].

Overall, there is a convergence of genetic and environmental risk factors, with TDP-43 being associated with general disease mechanisms in ALS and FTD. Furthermore, in addition to proteinopathy, the ALS–FTD spectrum is also associated with the presence of neuroinflammation and oxidative stress. In recent years, it has emerged that the transcription factor Nuclear Factor erythroid-derived 2-like 2 (herein referred to as NRF2, encoded by the *NFE2L2* gene) is an essential factor in modulating the expression of genes involved in antioxidant enzymes and biotransformation reactions, proteostasis, inflammation, and mitochondrial bioenergetics, among others [10,11]. Therefore, much emphasis has been placed on the possible use of elements of signaling for this transcription factor as a therapeutic target for diseases for which there is currently no treatment, such as ALS and FTD [12,13,14,15,16]. In basal conditions, there are low levels of NRF2 due to the action of an E3 ubiquitin ligase complex containing a substrate adaptor protein, Kelch-like ECH-associated protein 1 (KEAP1), which binds to and negatively regulates NRF2 [17]. However, in pathological conditions where there is a significant increase in oxidative stress, NRF2 signaling is induced through modifications of key cysteine residues in KEAP1, which induce conformational changes in the binding of NRF2–KEAP1 and prevent the degradation of NRF2. This allows the accumulation of newly synthesized NRF2, which can then translocate to the nucleus and bind to the antioxidant response element (ARE) sequence in the promoter regions of NRF2-dependent genes and recruit transcriptional machinery [18,19]. Many NRF2 activators have been identified. Among them, dimethyl fumarate (DMF) has been reliably demonstrated to act in the brain and to have neuroprotective effects [20,21], with the added value of being an already approved drug for relapsing–remitting multiple sclerosis, known as Tecfidera by Biogen [22].

In our working group, we have previously found that, in FTD-TAU models, hippocampal cells expressing human TAU (P301L) mutant protein produce the chemokine fractalkine (CX3CL1). In microglia, this activates AKT, inhibits glycogen synthase kinase-3β (GSK-3β), and upregulates the transcription factor NRF2/*NFE2L2* and its target genes, including heme oxygenase 1 [23]. Moreover, treatment with DMF, an inducer of transcription factor NRF2, could mitigate the neurodegenerative hallmarks in our FTD-TAU mouse model [21]. These results clearly demonstrate the relevance of NRF2 pharmacological modulation in FTD-TAU. However, in relation to the protein TDP-43, which is associated with both ALS and FTD pathologies, there is still limited evidence of the involvement of transcription factor NRF2. Therefore, in this study, we carried out an in-depth analysis of the transcriptional signature of NRF2 in animal models of FTP-TDP-43 and ALS-TDP-43^A315T^ as well as patient samples with sALS in order to confirm NRF2 as a pharmacological target for these diseases.

## 2. Materials and Methods

### 2.1. FTD Mouse Model: CaMKII-TDP-43

All animal experiments were conducted in 90-day-old FVB-N background FVB-N/CaMKII-TDP43 transgenic and nontransgenic mice that were bred in our animal facilities from initial breeders generously provided by Dr. Che-Kun J Sheen (The PhD Program for Neural Regenerative Medicine, Taipei Medical University, Taiwan) and previously characterized [24]. CaMKII-TDP-43 male mice and their wild-type animals were generated from homozygotic breeding.

### 2.2. ALS Mouse Model

All animal experiments were conducted in 90-day-old C57BL/6J background Prp-hTDP-43 (A315T) transgenic and nontransgenic littermate sibling mice that were bred in our animal facilities from initial breeders purchased from Jackson Laboratories (Bar Harbor, ME, USA) and previously characterized [25]. Prp-hTDP-43 (A315T) male mice and their wild-type littermate sibling were generated from heterozygotic breeding.

### 2.3. Animal Management, Sacrifice, and Sampling

Both colonies were housed in a CAI animal facility from UCM (ES-28079-0000086) in a room with controlled photoperiod (08:00–20:00 light) and temperature (22 ± 1 °C) with free access to high-fat jelly diet (DietGel Boost, ClearH20, Portland, ME, USA) specific for TDP-43 transgenic mice [25] and water. All animal experiments were conducted according to local and European rules (directive 2010/63/EU) and conformed to ARRIVE guidelines. They were approved by the ethical committees of our university and the regulatory institution (ref. PROEX 056/96).

In both colonies, animals were sacrificed at 90 days of age, and their brains and spinal cords were rapidly removed after decapitation. Brains to be used for histology were fixed for one day at 4 °C in fresh 4% paraformaldehyde prepared in 0.1 M phosphate-buffered saline (PBS), pH 7.4. Samples were cryoprotected by immersion in a 30% sucrose solution for a further day and finally stored at −80 °C for RNAscope and immunohistochemical analysis. Spinal samples (lumbar area) were used for biochemistry. They were collected and frozen by immersion in cold 2-methylbutane followed by storage at −80 °C until qPCR analysis.

### 2.4. Human Samples

Motor cortex samples (prerolandic and parietal cortex) from ALS patients and control subjects, including their clinical data (See Table 1), were provided by Biobank Banco de Tejidos CIEN (PT17/0015/0014), which is integrated in the Spanish National Biobanks Network. The data were processed following standard operating procedures with the appropriate approval of the ethics and scientific committees. Spinal cord samples from ALS patients and control subjects, including their clinical data (see Table 1), were provided by Intitut D’Investigacions Biomediques August Pi Sunyer (IDIBAPS). Immediately after brain extraction, the brain areas were quickly frozen fresh at −50 °C (in NOVEC) and immediately placed and stored at −80 °C. These frozen samples were used for RNA extraction and qRT-PCR analysis. The protocol used was similar to the one described in [26].

### 2.5. Analysis of mRNA Levels by Quantitative Real-Time PCR

Total RNA extraction, reverse transcription, and quantitative polymerase chain reaction (PCR) were done as detailed in the previous article [23]. Primer sequences are shown in Table 2. Data analysis was based on the ΔΔCT method with normalization of the raw data to housekeeping genes (Applied Biosystems, Waltham, MA, USA). All PCRs were performed in triplicates.

### 2.6. RNAscope

The brain sections (30 μm thickness) were cryoprotected in an antifreeze solution (30% glycerol + 30% ethylene glycol + 0.01 M PBS) for storage at −20 °C. The sections were removed from cryoprotectant and washed four times in TBS for 5 min each. Sections were incubated in Pretreatment 1 at RT until the bubbling ceased (40 min). The sections were washed four times in TBS for 1 min each. Floating sections were then placed in 1× Pretreatment 2 preheated to 99–100 °C and incubated for 12 min. Sections were immediately removed from Pretreatment 2 and placed in 0.5xTBS for mounting directly onto Superfrost^TM^ Plus adhesion microscope slides (one section per slide). The sections were dried at 60 °C overnight. The following day, sections were dipped in 100% EtOH and air-dried before creating a hydrophobic barrier around the tissue. Once the barrier was completely dry, the next step was carried out. Sections were incubated in Pretreatment 3 (2–3 drops/per section), taking care to completely cover the tissue. Then, the slides were incubated at 40 °C for 15 min before washing four times in H_2_O for 1 min each. All remaining incubation steps were conducted using this oven and tray system unless otherwise noted. RNAscope^®^ in situ hybridization assay was performed according to the manufacturer’s instructions (Advance Cell Diagnostics (ACD), Hayward, CA, USA). *Nqo1* (Cat. No. 317801-C1) and *Hmox1* (Cat. No. 498811-C2) RNAscope^®^ probes were purchased from ACD. In addition, the negative (Cat. No. 320871, ACD) and positive (Cat. No. 320881, ACD) control probes were applied and allowed to hybridize for 2 h at 40 °C. The amplification steps were performed according to the manufacturer’s directions. In between every amplification step, sections were washed with 2× wash buffer. Detection was performed using a mixture ratio of C1 to C2 solution of 1:60. Brain sections were rinsed with TBS three times and incubated for 5 min in TBS with DAPI solution for counterstained nuclei. RNAscope quantification was performed following the indications in [27]. The experiment was performed twice. In each experiment, a sagittal section of the brain was used, where the prefrontal cortex for the FTD animal model or the motor cortex for the ALS transgenic model was located. In each animal (*n* = 3–4), 2 pictures were taken of the selected field in which quantification was performed. Transcript expression was analyzed per area using Fiji analysis.

### 2.7. Immunoblotting

Whole-tissue lysates were prepared in RIPA buffer (25 mM Tris-HCl pH 7.6, 150 mM NaCl, 1 mM EGTA, 1% Igepal, 1% sodium deoxycholate, 0.1% SDS, 1 mM PMSF, 1 mM Na_3_VO_4_, 1 mM NaF, 1 μg/mL aprotinin, 1 μg/mL leupeptin and 1 μg/mL pepstatin). Whole-cell lysates containing 50 μg of whole proteins from prefrontal cortex and 30 μg of whole proteins from spinal cord were loaded for SDS-PAGE electrophoresis. Immunoblots were performed as described in [23]. The primary antibodies used are described in Table 3.

### 2.8. Measurement of Thiobarbituric Acid Reactive Substances (TBARs)

Lipid peroxidation was determined as the formation of thiobarbituric acid reactive substances (TBARS) according to a previous report [28]. Frozen tissue from human lumbar spinal cord was weighted and homogenized in 200 µL PBS, and 400 μL of TBA reagent (0.375 g TBA, 7.5 g trichloroacetic acid, and 2.54 mL HCl) was added before incubating at 95 °C for 30 min. A pink chromophore was produced in samples in direct relation to the amount of peroxidized products. Samples were then kept in ice for 5 min and centrifuged at 3000× *g* for 15 min. The optical density of the supernatants was measured in a spectrometer at 532 nm. The amount of TBARS (mostly malondialdehyde (MDA)) was calculated by interpolation of values in a constructed MDA standard curve with 1,1,3,3-tetrametoxypropane, and the results were expressed as nanomoles of MDA per mg of tissue.

### 2.9. Statistical Analyses

Data are presented as mean ±SEM. To determine the statistical test to be used, we employed GraphPad Instat 3, which includes analysis of the data to normal distribution via the Kolmogorov–Smirnov test. In addition, statistical assessments of differences between groups were analyzed (GraphPad Prism 5, San Diego, CA, USA) by unpaired Stu-dent’s *t*-tests when normal distribution and equal variances were fulfilled or by the nonparametric Mann–Whitney *U*-test. One- and two-way ANOVA with post hoc Newman–Keuls test or Bonferroni’s test were used as appropriate.

## 3. Results

TDP-43 protein is implicated in both FTD and ALS, although its contribution in the pathogenesis varies from one to the other depending on the origin of changes in TDP-43 (gene mutations, protein covalent modifications, subcellular location, etc.). Therefore, in this study, we used two different transgenic mouse models based on TDP-43 overexpression resembling either FTD or ALS to assess the status of the NRF2 signaling pathway. We mainly focused on its antioxidant function and also analyzed the expression levels of downstream genes/proteins such as HO-1 and NQO1. Previously, we have observed that, in sALS lymphoblast, there is an increase in NRF2/HO-1/NQO1 compared to control samples [29]. Therefore, we wanted to focus our attention on these two antioxidant enzymes. Finally, we determined the significance of these findings in human motor cortex and spinal cord samples to elucidate the potential use of NRF2 signaling as a therapeutic target in ALS.

### 3.1. TDP-43 Overexpression Decreased NQO1 Expression in a Model of FTD

**Figure 1:**
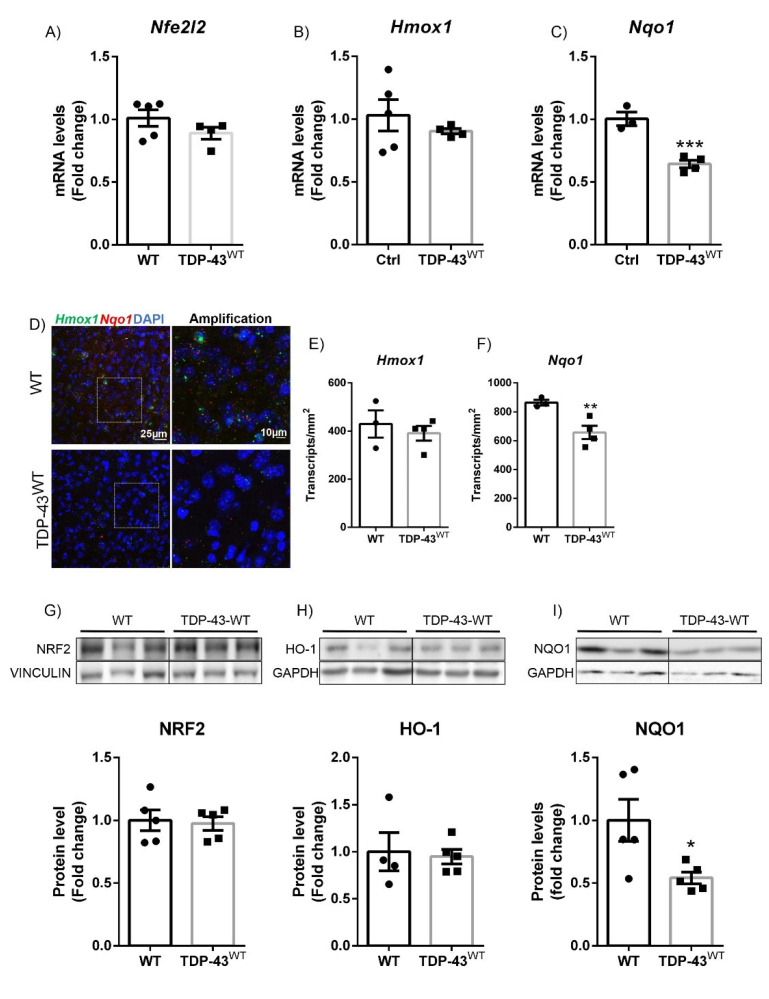
TDP-43-dependent FTLD transgenic mouse model showed decreased NQO1 expression in the prefrontal cortex. Quantitative real-time PCR determination of mRNA levels of (**A**) *Nfe2l2*, (**B**) *Hmox1*, and (**C**) *Nqo1*. All genes were normalized by *Tbp* (TATA box binding protein) mRNA levels; *n* = 4–5 samples ±SEM. Asterisks denote significant differences at *** *p* < 0.001 when comparing the indicated group to the wild-type mice according to the Student’s *t*-test. (**D**) RNAscope ISH labelling of *Hmox1* (green) and *Nqo1* (red) in prefrontal cortex of 90-day-old CaMKII-TDP-43-WT mice. Quantification of (**E**) *Hmox1* and (**F**) *Nqo1* expression using Fiji analysis. Asterisks show significant differences at ** *p* < 0.01 when comparing groups according to the Student’s *t*-test. Protein levels of (**G**) NRF2, (**H**) HO-1, and (**I**) NQO1 were analyzed by immunoblotting and their respective protein quantifications; *n* = 4–5 samples per experimental group ±SEM. Asterisks show significant differences at * *p* < 0.05 when comparing groups according to the Student’s *t*-test.

To gain more insight into the role of NRF2 signaling in a TDP-43-based FTD model, we analyzed the expression pattern of the NRF2 antioxidant pathway in the prefrontal cortex of CaMKII-TDP-43-WT from 90-day-old transgenic mice compared to wild-type. These mice overexpressed TDP-43 only in the forebrain, mimicking the characteristic phenotype of FTLD with ubiquitin-positive inclusions (FTLD-U) [24] without degeneration of the spinal cord. Our results showed that *Nfe2l2* and *Hmox1* mRNA expression levels (Figure 1A,B) as determined by qPCR were similar between genotypes. Unchanged *Hmox1* mRNA expression levels were confirmed by RNAscope (Figure 1D,E). Comparable results were obtained at the protein level (Figure 1G,H), with NRF2 and HO-1 levels revealing no difference between genotypes. However, in the case of *Nqo1*, we observed a significant decrease in mRNA expression levels as measured by qPCR and corroborated by RNAscope (Figure 1C,D,F). Furthermore, this decrease in mRNA levels was reflected at the protein level, which showed a reduction of approximately 50%. These data indicated that TDP-43 overexpression in the prefrontal cortex of transgenic mice did not induce significant changes in NRF2 antioxidant signaling, although there was a significant decrease in NQO1 levels. This specific alteration of NQO1 levels due to TDP-43 overexpression may be related to the RNA binding and translation control functions of both TDP-43 and NQO1 proteins. TDP-43 has been found to be involved in mRNA splicing, stability, metabolism, and translation [30]. More recently, it has been shown that, apart from its involvement in the cellular redox switch, NQO1 is an RNA-binding protein that is involved in the control of translation [31].

### 3.2. A315T Mutation in TDP-43-Induced NRF2 Signaling in a Model of Amyotrophic Lateral Sclerosis (ALS)

Apart from the implication of TDP-43 in the pathology of FTLD, mutations of this protein are associated with familial forms of ALS [32]. Therefore, we analyzed how the A315T mutation in the TDP-43 protein could influence the NRF2 antioxidant signaling pathway in a transgenic mouse model for ALS at a symptomatic stage [33].

**Figure 2:**
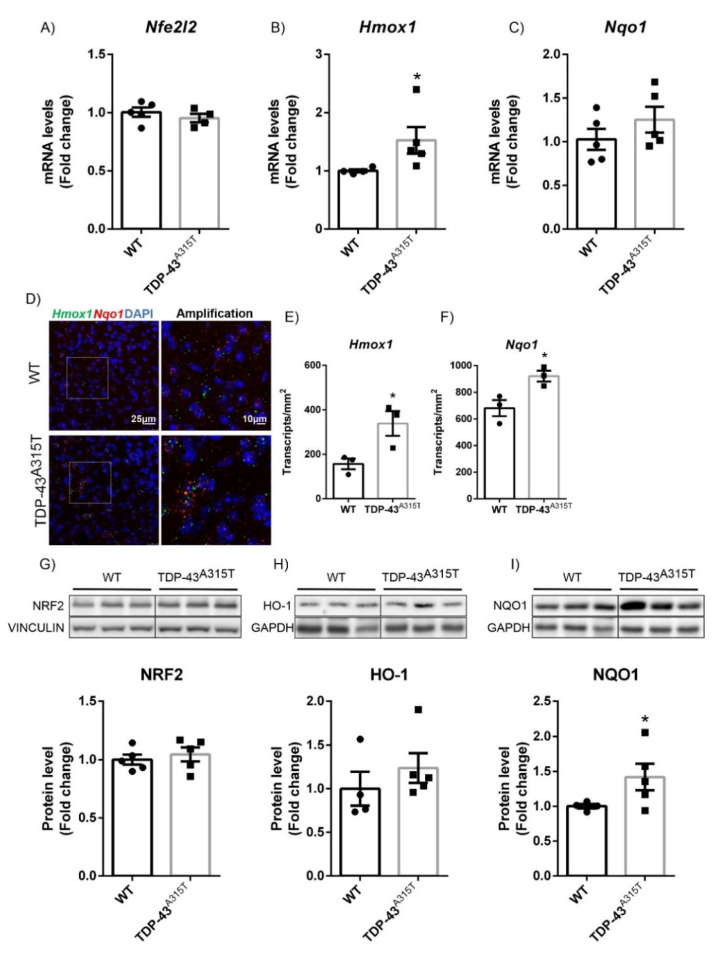
TDP-43^A315T^ transgenic mice showed increased HO-1 and NQO1 expression in motor cortex. Quantitative real-time PCR determination of mRNA levels of (**A**) *Nfe2l2*, (**B**) *Hmox1*, and (**C**) *Nqo1*. All genes were normalized by *Tbp* (TATA box binding protein) mRNA levels; *n* = 4–5 samples ±SEM. Asterisks denote significant differences at * *p* < 0.05 when comparing the indicated group to the wild-type mice according to the Student’s *t*-test. (**D**) RNAscope ISH labelling of *Hmox1* (green) and *Nqo1* (red) in prefrontal cortex of 90-day-old TDP-43^A315T^ transgenic mice. Quantification of (**E**) *Hmox1* and (**F**) *Nqo1* expression using Fiji analysis. Asterisks show significant differences at * *p* < 0.05 when comparing groups according to the Student’s *t*-test. Protein levels of (**G**) NRF2, (**H**) HO-1, and (**I**) NQO1 were analyzed by immunoblotting and their respective protein quantifications; *n* = 4–5 samples per experimental group ±SEM. Asterisks show significant differences at * *p* < 0.05 when comparing groups according to the Student’s *t*-test.

These animals expressed TDP-43^A315T^ under the control of the mouse prion protein (PrP), showing TDP-43^A315T^ overexpression in the brain and spinal cord [25]. First, we assessed the status of NRF2 pathway in the motor cortex of 90-day-old TDP-43^A315T^ transgenic mice. These animals have been found to show energy deficiency and oxidative stress in the motor cortex [34], suggesting that the NRF2 pathway could be compromised in these mice at this state of disease progression. Our results showed that *Nfe2l2* mRNA expression levels (Figure 2A) as determined by qPCR were similar between genotypes, as would be expected due to the fact that the NRF2 pathway is mainly regulated at the protein level [19]. However, at the NRF2 protein level (Figure 2G), there were no significant differences between genotypes. Nevertheless, in the case of *Hmox1* and *Nqo1*, we observed significantly increased mRNA expression levels as measured by qPCR (Figure 2B,C, respectively) (with a trend observed in the case of *Nqo1*) and corroborated by RNAscope in both cases (Figure 2D–F). These increased mRNA levels were confirmed at the protein level, where there was a significant increase in NQO1 protein expression (Figure 2I) and a trend for HO-1 (Figure 2H). These data indicated that TDP-43^A315T^ overexpression in the motor cortex of transgenic mice did induce a significant increase in the expression pattern of the NRF2 pathway.

**Figure 3:**
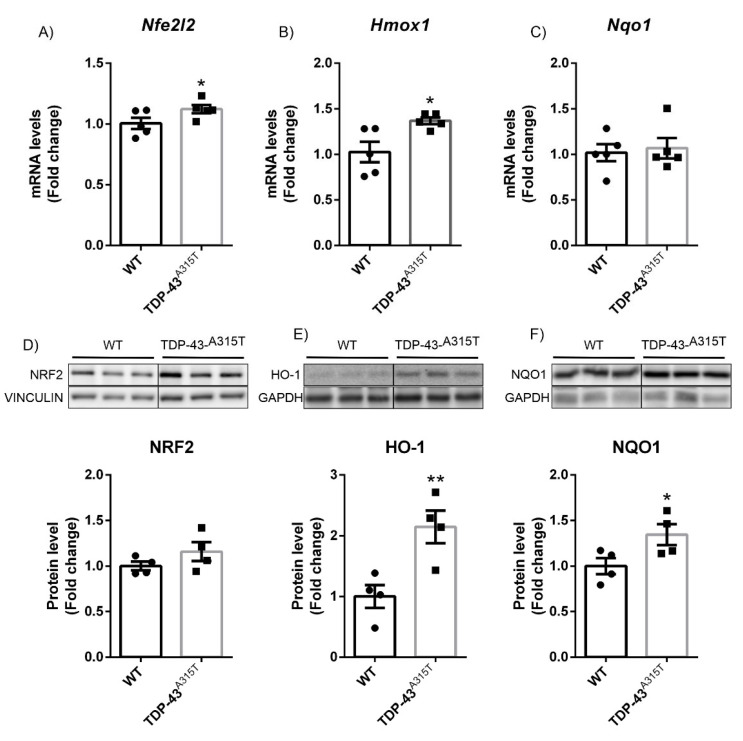
TDP-43^A315T^ transgenic mice showed increased HO-1 and NQO1 expression in lumbar spinal cord. Quantitative real-time PCR determination of mRNA levels of (**A**) *Nfe2l2*, (**B**) *Hmox1*, and (**C**) *Nqo1*. All genes were normalized by *Tbp* (TATA box binding protein) mRNA levels; *n* = 4–5 samples ±SEM. Asterisks denote significant differences at * *p* < 0.05 and ** *p* < 0.01 when comparing the indicated group to the wild-type mice according to the Student’s *t*-test. Protein levels of (**D**) NRF2, (**E**) HO-1, and (**F**) NQO1 were analyzed by immunoblotting and their respective protein quantifications; *n* = 4 samples per experimental group ±SEM. Asterisks show significant differences at * *p* < 0.05 when comparing groups according to the Student’s *t*-test.

These mice have been found to show features of ALS and FTLD in the spinal cord [25,33]. Therefore, we also analyzed NRF2 signature in lumbar spinal cord samples. Our results showed that *Nfe2l2* mRNA expression levels (Figure 3A) as determined by qPCR were slightly increased in TDP-43^A315T^ overexpressing mice. This small difference did not become statistically significant at the protein level (Figure 3D). Nevertheless, in the case of *Hmox1* and *Nqo1*, we observed a significant increase in mRNA expression levels as measured by qPCR (Figure 3B,C, respectively) (with a trend observed in the case of *Nqo1*). Unfortunately, the spinal cords of these mice were not processed in a way that RNAscope analysis could be performed. At the protein level, there was a significant increase in expression of HO-1 and NQO1 in the TDP-43^A315T^ transgenic mice in comparison to their WT littermates. These data indicated that, similar to what was observed in the motor cortex, TDP-43^A315T^ overexpression induced a significant increase in the expression pattern of the NRF2 signature in the spinal cord. These data are in agreement with the fact that overexpression of TDP-43^A315T^ produces oxidative stress, which induces activation of the NRF2 antioxidant pathway.

### 3.3. Altered NRF2 Signaling Pathway in ALS Human Samples

Finally, we assessed the status of NRF2 signaling in human motor cortex and lumbar spinal cord samples obtained from controls and sporadic ALS (sALS) patients. There is a previous study where the levels of expression of the NRF2 protein in samples from patients with ALS were analyzed [35] but not its antioxidant signaling pathway. Therefore, we wanted to determine in greater detail not only the levels of NRF2 protein expression (which in some cases may not be altered) but also genes dependent on this transcription factor, such as *HMOX1* and *NQO1*.

**Figure 4:**
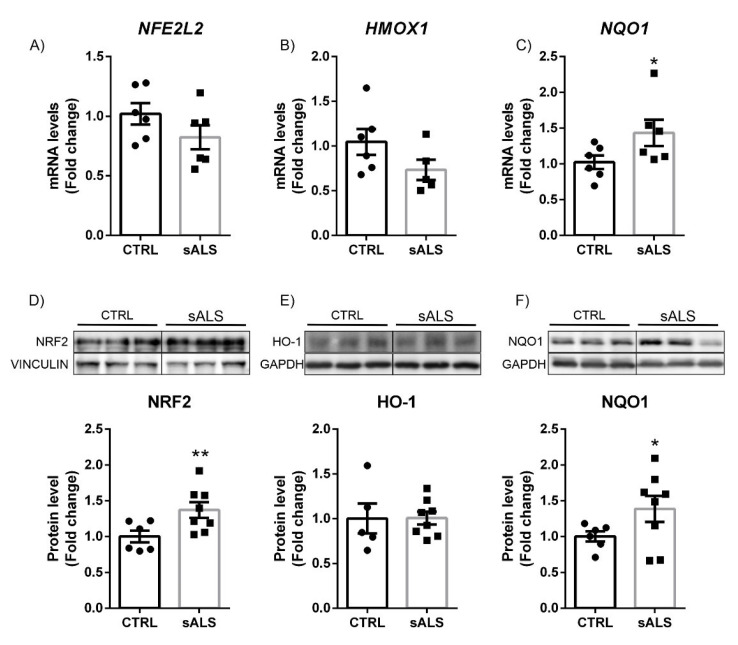
Increased NRF2 and NQO1 protein levels in the motor cortex in sALS patients. Quantitative real-time PCR determination of mRNA levels of (**A**) *NFE2L2*, (**B**) HMOX1, and (**C**) NQO1. All genes were normalized by *TBP* (TATA box binding protein) mRNA levels; *n* = 6 samples ±SEM. Asterisks denote significant differences at * *p* < 0.05 when comparing sALS to control patients according to the Student’s *t*-test. Protein levels of (**D**) NRF2, (**E**) HO-1, and (**F**) NQO1 were analyzed by immunoblotting and their respective protein quantifications; *n* = 6–8 samples per experimental group ±SEM. Asterisks show significant differences at * *p* < 0.05 and ** *p* < 0.001 when comparing groups according to the Student’s *t*-test.

In the motor cortex, mRNA expression levels of *NFE2L2* and *HMOX1* barely changed between controls and patients with sALS (Figure 4A,B), although there was a significant increase in *NQO1* mRNA expression levels (Figure 4C). These results were similar to those observed in the mouse model of ALS with overexpression of TDP-43^A315T^. Moreover, when we analyzed protein levels of this pathway, we found a significant increase in NRF2 and NQO1 protein levels (Figure 4D,F), indicating that NRF2 signaling was overactivated in the motor cortex of sALS. However, in the case of the HO-1 enzyme, we did not observe differences between the controls and the sALS (Figure 4E).

**Figure 5:**
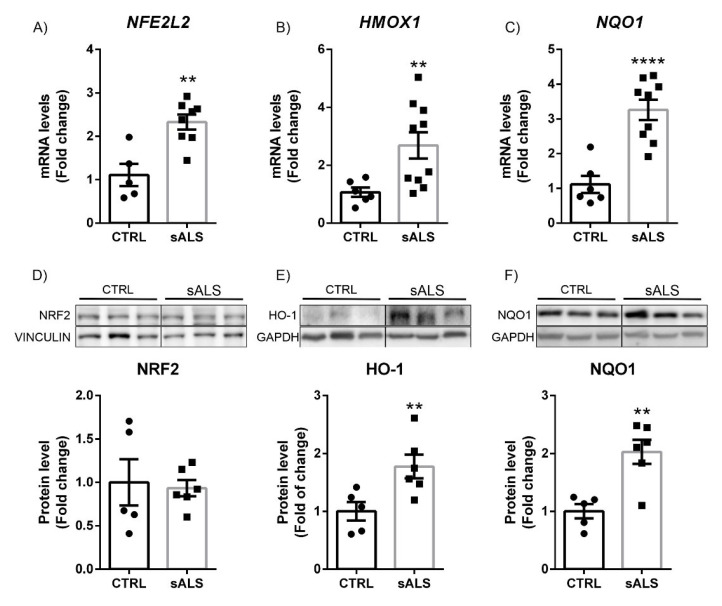
Increased HO-1 and NQO1 protein levels in lumbar spinal cord in sALS patients. Quantitative real-time PCR determination of mRNA levels of (**A**) *NFE2L2*, (**B**) *HMOX1*, and (**C**) *NQO1*. All genes were normalized by *TBP* (TATA box binding protein) mRNA levels; *n* = 6–8 samples ±SEM. Asterisks denote significant differences at ** *p* < 0.001 and **** *p* < 0.0001 when comparing sALS to control patients according to the Student’s *t*-test. Protein levels of (**D**) NRF2, (**E**) HO-1, and (**F**) NQO1 were analyzed by immunoblotting and their respective protein quantifications; *n* = 5–6 samples per experimental group ±SEM. Asterisks show significant differences at ** *p* < 0.001 when comparing groups according to the Student’s *t*-test.

**Figure 6:**
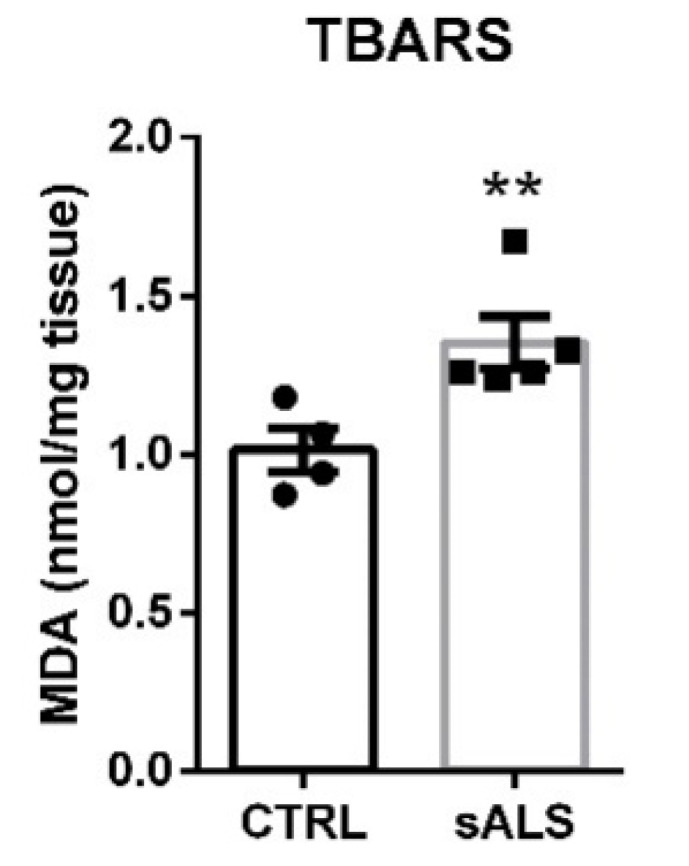
Increased TBARS levels in lumbar spinal cord in sALS patients. Thiobarbituric acid reactive substances (TBARs) representing malondialdehyde (MDA), resulting from oxidation of lipid substrates. Dots indicate the mean of *n* = 4 (controls) and *n* = 5 (sALS) samples ±SEM. Asterisk denote significant differences at ** *p* < 0.01 when comparing both groups according to the Student’s *t*-test.

Finally, we also investigated the status of the NRF2 signaling pathway in the spinal cord of sALS patients, which is the one mainly affected by the neurodegenerative process in this pathology. Interestingly, our data showed a statistically significant increase in mRNA expression levels of *NFE2L2* (Figure 5A), *HMOX1* (Figure 5B), and *NQO1* (Figure 5C), which may well be a consequence of the observed increase in oxidative stress in this tissue induced by ALS-associated neurodegeneration [36,37]. At the protein level, we did not observe a significant increase in the levels of NRF2 (Figure 5D) due to the great variability presented by the subjects in the control group. However, a highly significant increase was observed in the expression levels of HO-1 (Figure 5E) and NQO1 (Figure 5F), supporting the idea that increased oxidative stress in the spinal cord activated the antioxidant machinery regulated by the NRF2 pathway. To confirm this, we employed the thiobarbituric acid reactive substance (TBARS) assay as a method to detect lipid oxidation. This assay measures malondialdehyde (MDA), which is a split product of endoperoxide of unsaturated fatty acids resulting from oxidation of lipid substrates. Analysis of TBARS levels indicates that the lumbar spinal cord of sALS showed increased lipid peroxidation in comparison to control samples (Figure 6).

## 4. Discussion

Although much has been written and speculated about the role of the NRF2 pathway in ALS and its possible use as a therapeutic target, there is little experimental data on the status of this pathway in both ALS and its associated FTD spectrum pathology. Therefore, in this work, we wanted to carry out in-depth analysis of this signaling pathway in both transgenic mouse models (ALS and FTD) and in human samples from patients with sALS versus controls. Our results indicate that, in patients with sALS, there is an increase in the NRF2 pathway in both the motor cortex and the lumbar spinal cord. Similar data obtained in the murine ALS model caused by overexpression of TDP-43^A315T^ confirmed such significant increase in the signaling pathway for this transcription factor at the symptomatic state of disease progression.

On the other hand, moving to the murine model of FTD, also based on the TDP-43 protein, we observed a decrease in the expression of NQO1 without any other relevant alterations in the NRF2 pathway itself. These results suggest that, depending on the specific condition recapitulated in the experimental models of the ALS–FTD spectrum, the specific pathological alterations may or may not modify the NRF2 pathway.

Previous analysis conducted in our laboratory with samples of sALS lymphoblasts demonstrated an increase in oxidative stress (ROS and lipid peroxidation), which was accompanied by a significant increase in the NRF2 antioxidant signaling pathway [29,38]. The data obtained in this study confirm what we have found in postmortem samples from patients with sALS, namely, a significant increase in the NRF2 antioxidant pathway in both the lumbar spinal cord and the motor cortex. These data in turn confirm the usefulness of the patient lymphoblast cell model as it recapitulates many features of affected motor neurons, such as alterations in TDP-43 homeostasis, oxidative stress, and NRF2 changes [39], and can be used as a suitable cell model platform for preclinical evaluation. Furthermore, the relevance of the NRF2 pathway in ALS pathology is also reinforced by the fact that one haplotype in *NFE2L2*, which includes three functional promoter SNPs associated with increased protein expression of NRF2, was associated with delayed disease onset of 4 years per allele [40]. An important question that still remains to be clarified in future experiments relates to whether the cell type NRF2 activation occurs in neuron, astrocyte, or microglia. It has been found that, in astrocytes, there is poor mitochondrial respiration but high reactive oxygen species (ROS) production, whereas neurons have high mitochondrial respiration and low ROS production [41,42]. Therefore, it is likely that the increase in NRF2 signaling pathway predominantly takes place in astrocytes/microglia, as is the case in other pathologies, such as Parkinson’s and Alzheimer’s diseases [20,23,43]. However, it was shown that overexpression of APP resulted in the downregulation of NRF2 levels among mitochondrial-related proteins, with the inactivation of NRF2 in primary neuron culture impairing the activity of the electron transport chain complexes, thereby decreasing the efficacy of oxidative phosphorylation [44]. Preliminary experiments from our laboratory (unpublished data) suggest that overexpression of TDP-43 (both the wild-type protein and with the A315T mutation) in the motor neuron-like cell line NSC-34 induces NRF2/HO-1 expression. However, regardless of where overexpression of the NRF2 antioxidant pathway occurs, this increase in the NRF2 pathway does not appear to be enough to restore cellular homeostasis. Therefore, greater increase would be necessary through therapeutic strategies such as DMF, which is currently already used in clinical practice for patients with multiple sclerosis to attenuate/delay neurodegeneration [20,21,45]. In this respect, it has been shown that overexpression of NRF2 in astrocytes in a murine model of ALS significantly delayed onset and extended survival [46], whereas restricted NRF2 overexpression in neurons was able to delay disease onset but failed to extend survival [47]. These findings validate NRF2 as a target for induction in glial cells and as a viable therapeutic tool for ALS.

Previous data in human samples from patients with ALS indicated a decrease in *NFE2L2* mRNA and protein levels, which is completely opposite to the results obtained in our study [35]. In our study, we used *n* = 6–8 samples per experimental group, whereas the sample size was considerably lower in the previous study (*n* = 3). Considering the intrinsic variability that exists between different patients, an too small sample size can lead to inaccurate conclusions. On the other hand, confusion regarding the correct migratory pattern of NRF2 raises doubts about the accuracy and reproducibility of the published results. Although the biologically relevant molecular weight of NRF2 has been described as ∼95–110 kilodalton (kDa), many manuscripts shown the predicted ∼55–65 kDa, invalidating the presented data [48]. In our laboratory, we tested antibodies against NRF2 from various commercial companies until we found one in which we saw good specificity. In relation to ALS patients, we must bear in mind the heterogeneity of phenotypes, which are based on the heterogeneity of the proteins involved. Studies carried out in cell or mouse models with mutations in superoxide dismutase 1 (SOD1) indicate a decrease in the NRF2 signaling pathway [49,50]. These data are consistent with data obtained in lymphoblasts from patients with familial ALS derived from mutations in SOD1 [29,39]. Therefore, the pharmacological modulation of NRF2 as a therapeutic strategy for ALS should be personalized based on the molecular alterations displayed by different types of patients.

One of the most characteristic neuropathological features of ALS is the presence of neuronal cytoplasmic inclusions of TDP-43 protein in the degenerating motor neurons [51]. The discovery that TDP-43 is present in cytoplasmic aggregates in both ALS and FTLD-U provides evidence that the two disorders may share a common underlying mechanism. Remarkably, approximately 20% of patients with ALS also develop FTD, and approximately 15% of FTD patients also develop ALS [52,53]. Therefore, we studied the role of NRF2 associated with TDP-43 in the development of ALS or FTD in mouse models of the diseases. Although the murine models are not comparable to each other due to the differences in the degree of expression of the proteins and the different promoters, they can give us an idea of the differences that exist between the two pathologies within the same spectrum. For the ALS model, we used transgenic mice that overexpressed TDP-43^A315T^, which is a well-established model [25,38,54]. Within these mice, this mutant form of human TDP-43 showed protein aggregates in the frontal cortex and spinal motor neurons. Similar to human samples, in these TDP-43^A315T^ transgenic mice, we observed an increase in NRF2 signature in the frontal cortex and lumbar spinal cord (Figure 2 and Figure 3). Comparable results were also obtained for TDP-43^M337V^ postsymptomatic fibroblasts compared to controls and for the spinal cord of TDP-43^Q33IK^ transgenic mice, where a significant increase in HO-1 and NQO1 was observed [55]. In the case of the FTD model, we used a model of overexpression of the TDP-43^WT^ protein in the forebrain under the CaMKII promoter, which leads to the appearance of a phenotype that resembles FTLD-U [24]. At the stage of analysis, in the prefrontal cortex of these mice, we only observed a significant decreased expression of NQO1, at both mRNA and protein levels (Figure 1), that cannot be attributed to alterations in the NRF2 pathway.

Besides its involvement in cellular redox control, emerging data indicate that NQO1 is an RNA-binding protein and is involved in the control of translation. As TDP-43 regulates RNA metabolism, and trafficking, and interacts with several proteins that are implicated in its RNA-processing functions, it cannot be ruled out that the overexpression of TDP-43 specifically modulates NQO1. Future experiments will be necessary to determine the interrelationship between both proteins. Also, in further experiments, it would be interesting to determine the status of NRF2 signature, or specifically NQO1, in samples from patients with TDP-43 dependent FTD.

In addition to TDP-43, mutations in microtubule-associated protein TAU (MAPT) have been found to be the most common cause of familial FTD [56]. Previous work from our lab demonstrated that, in a mouse model based on stereotaxic delivery in the hippocampus of an adeno-associated viral vector for expression of TAU^P301L^, a mutated form of TAU associated with FTD, NRF2 signaling pathway was increased in astrocytes and microglia and treatment with the NRF2 inducer DMF provided neuroprotection [21,23]. These data further suggest the need for personalized treatment in patients with ALS and FTD based on their genetic background.

## 5. Conclusions

In summary, our results show that the NRF2 antioxidant pathway is differently affected in ALS and FTD. Our data clearly demonstrate that the NRF2 signaling pathway is increased in sALS patients to combat the increase in oxidative stress in symptomatic stages, but it is not sufficient to restore cellular homeostasis. Therefore, therapies that can potentiate the action of the NRF2 pathway could be beneficial for patients suffering from ALS. Further work is needed to establish a more specific and personalized pharmacological treatment for ALS and FTD patients.

## Figures and Tables

**Table 1:** List of human samples used in this study.

Patient ID	Clinic Presentation	Age	Gender	Samples
BCPA00870	ALS	71	M	FROZEN MOTOR CX
BCPA00746	ALS	67	F	FROZEN MOTOR CX
BCPA00694	ALS	77	M	FROZEN MOTOR CX
BCPA00658	ALS	77	M	FROZEN MOTOR CX
BCPA00493	ALS	79	F	FROZEN MOTOR CX
BCPA00405	ALS	80	F	FROZEN MOTOR CX
BCPA00347	ALS	67	F	FROZEN MOTOR CX
BCPA00481	ALS	71	F	FROZEN MOTOR CX
BCPA00364	Control	43	M	FROZEN MOTOR CX
BCPA00537	Control	74	F	FROZEN MOTOR CX
BCPA00587	Control	83	F	FROZEN MOTOR CX
BCPA00662	Control	58	F	FROZEN MOTOR CX
BCPA00849	Control	61	F	FROZEN MOTOR CX
BCPA00972	Control	83	M	FROZEN MOTOR CX
381	ALS	72	M	FROZEN SPINAL CORD
1216	ALS	78	M	FROZEN SPINAL CORD
1344	ALS	60	F	FROZEN SPINAL CORD
1407	ALS	65	F	FROZEN SPINAL CORD
1872	ALS	66	M	FROZEN SPINAL CORD
1971	ALS	71	F	FROZEN SPINAL CORD
1694	Control	58	M	FROZEN SPINAL CORD
1697	Control	78	M	FROZEN SPINAL CORD
1818	Control	78	M	FROZEN SPINAL CORD
1858	Control	83	F	FROZEN SPINAL CORD
1888	Control	93	F	FROZEN SPINAL CORD
1937	Control	83	F	FROZEN SPINAL CORD
1949	Control	86	M	FROZEN SPINAL CORD

**Table 2:** List of primers used in this study.

Gene Product	Forward Primer	Reverse Primer
*β-ACTIN*	5′ TCCTTCCTGGGCATGGAG 3′	5′ AGGAGGAGCAATGATCTTGATCTT 3′
*HMOX1*	5′ TGCTCAACATCCAGCTCTTTGA 3′	5′ GCAGAATCTTGCACTTTGTTGCT 3′
*Hmox1*	5′ CACAGATGGCGTCACTTCGTC 3′	5′ GTGAGGACCCACTGGAGGAG 3′
*NFE2L2*	5′CCCGAAGCACGCTGAAGGCA 3′	5′ CCAGGCGGTGGGTCTCCGTA 3′
*NQO1*	5′ GTTCATAGGAGAGTTTGCTT 3′	5′ TAGAACCTCAACTGACACTT 3′
*Nfe2l2*	5′ CCCGAAGCACGCTGAAGGCA 3′	5′ CCAGGCGGTGGGTCTCCGTA 3′
*Nqo1*	5′ GGTAGCGGCTCCATGTACTC 3′	5′ CATCCTTCCAGGATCTGCAT 3′
*TBP*	5′ TGCACAGGAGCCAAGAGTGAA 3′	5′ CACATCACAGCTCCCCACCA 3′

**Table 3:** List of antibodies used in this study.

Antibody	Source	Catalog Number	Dilution
GAPDH (6C5)	Santa Cruz Biotechnologies ()	sc-32233	1:1000
HO-1	Millipore	AB1284	1:1000
IBA1	Wako	019-19741	1:500
LAMIN B	Santa Cruz Biotechnologies	sc-6217	1:1000
NRF2	Abyntek	AJ1555a	1:2000
NQO1	Abcam	ab2346	1:2000
VINCULIN (N3C1)	Gentex	GTX109749	1:1000

## Data Availability

Not applicable.
